# Excessive Work Hours of Physicians in Training in El Salvador: Putting Patients at Risk

**DOI:** 10.1371/journal.pmed.0040205

**Published:** 2007-07-17

**Authors:** Kenneth R Fernández Taylor

*Let us emancipate the student, and give him time and opportunity for the cultivation of his mind, so that in his pupilage he shall not be a puppet in the hands of others, but rather a self-relying and reflective being.*

—Sir William Osler, Aphorisms from His Bedside Teachings and Writings

Recent studies involving physicians in training have shown that excessive work hours are associated with an increased rate of medical errors and adverse events. Studies of surgical residents found that they made up to twice the number of technical errors in the performance of simulated laparoscopic surgical skills after working overnight than after a night of sleep [1,2]. A landmark study of interns working in an intensive care unit found that they made substantially more serious medical errors when they worked frequent shifts of 24 hours or more than when they worked shorter shifts [3]. A national prospective study of interns in the United States found that extended-duration work shifts (defined as at least 24 continuous hours at work) were associated with an increased risk of significant medical errors, adverse events, and attentional failures [4]. According to the Institute of Medicine [5], preventable medical errors are responsible for the death of up to 98,000 people in the US every year, accounting for a higher mortality than car accidents, breast cancer, or AIDS. Although it is not known how many of these medical errors were due to fatigue, it is now acknowledged that fatigue associated with the excessive work hours of physicians in training is an important cause of medical errors. Hence, the issue of excessive work hours of physicians in training is becoming a public health priority in many countries worldwide.

In the US and Europe, concerns about the effects of excessive work hours upon patient safety led to legislation on duty-hour restrictions [6–8], and more change seems to be on the way [9]. For example, the European Working Time Directive limits junior doctors' working hours to 56 hours per week (to be reduced to 48 hours by 2009) [8], but many countries, including El Salvador, have taken no steps at all to limit work hours of medical trainees. The current European maximum of 56 hours per week is less than half the time a physician in training works in El Salvador. Many other Latin American countries also force their junior doctors to work similarly excessive hours. In this article, I share my personal experience of a typical working week, discuss the consequences of excessive work hours, and suggest ways to solve the problem.

**Figure:**
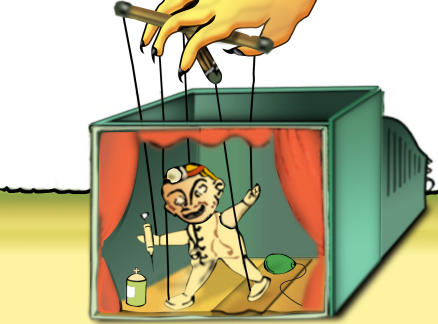
(Illustration: Luis Chan Chan)

## A Rite of Passage: A Workweek of 123 Hours

In El Salvador, physicians in training in their seventh year (out of the eight-year medical degree course) are called interns. An intern plays the dual role of medical student and institutional employee, hired for 365 days of compulsory work at a national hospital. The trial by fire begins on January 1st and ends on December 31st; interns work seven days a week, with almost no days scheduled for rest. Interns earn a monthly salary of $US362 for more than 480 hours of work. Hence the hourly wage is 75 cents. Many interns go completely unpaid.

If you're an intern, you arrive at the hospital at 5:00 a.m. and leave at 5:00–6:00 p.m. on weekdays; on weekends you arrive at 7:00 a.m. and leave at 12:00 p.m. You have a 36-hour shift every third day. That means that if you have a shift on Sunday, you'll also have a shift on Wednesday and Saturday that same week, without a day off for recovery in between. If you're lucky, you might be able to sleep one or two hours during a shift and have 20–30 minutes for meals. This working schedule adds up to about 123 hours in a week (a week has 168 hours in total), with only 3-5 hours per week dedicated for educational activities. Furthermore, most of the time physicians in training work without any kind of supervision by attending physicians. It is no wonder that the excessive work hours of trainee physicians have been called the Achilles' heel of the medical profession [10].

## The Workload

The excessive work hours of physicians in training in public hospitals are coupled with an enormous workload. For instance, the national hospital where I did my internship is a small second-level hospital of 222 beds. However, it is the only hospital for the Department of La Libertad, which has a population of more than 620,000 people. In 2006, 19,838 patients were hospitalized, 44,671 emergencies and 4,367 deliveries were attended, and 5,542 major surgeries were performed. There are only 125 nurses, 70 attending physicians, 40 residents, and 44 interns for the entire clinical section of the hospital, including the operating and delivery rooms. During a pediatrics night shift, for example, there is only one resident, one intern, and two nurses assigned for the ward (which houses around fifty children). During the night, there may be over twenty admissions, whom the intern will have to admit alone. The intern also has to take the vital signs of every patient at least two to three times during the night, among many other duties.

This inhumane approach to medical training has been witnessed by over 85 medical students from the International Federation of Medical Students' Associations from the US, Europe, and other countries. These students travelled to El Salvador as part of a medical student exchange scheme, and have criticized the pernicious working conditions their fellow students must face in El Salvador.

## Consequences of Excessive Working Hours

There are several harmful consequences of the excessive work hours of physicians in training, which may be divided into short term and long term consequences. In the short term, such work hours lead to sleep deprivation [11], and the associated fatigue that threatens patient safety [12]. Lack of sleep causes neurobehavioral impairments such as decrements in attention and vigilance as well as an inability to recognize the degree of one's impairment in performance after a night on call [13]. Dawson and Reid found that “relatively moderate levels of fatigue (after 17 hours of sustained wakefulness) impair performance to an extent equivalent to or greater than is currently acceptable in western industrialized countries for alcohol intoxication” [14]. Attentional failures associated with lack of sleep can occur while working directly with patients [15]. Such failures are related to the inhibition of cognitive processes such as memory and insight formation [16]. Extended work shifts by interns are also associated with an increased risk of motor vehicle crashes [17].

In the long term, excessive work hours and the associated sleep deprivation can increase the risk of burnout, anxiety, depression, and suicide [18]. Suicide has been reported as the second leading cause of death among medical students [19]. In their study of mood changes in medical interns over the course of their one-year internship, Lisa Bellini and colleagues found that enthusiasm at the beginning of internship soon gave way to depression, anger, and fatigue [20].

## Action Is Needed

The problem of excessive work hours of physicians in training in El Salvador has political and economic roots that need to be addressed by politicians and public health policy makers. Currently, health care is not given the place it deserves on the political agenda. The Ministry of Health receives insufficient resources from the government to serve the needs of the public network of hospitals (where the poor seek medical care). Only 1.62% of the country's gross domestic product is assigned to public sector health care, which serves 85% of the population, whereas 6.38% is assigned to the private health care sector [21]. Of the limited public sector resources, a large proportion is spent on salaries. For example, at Benjamin Bloom National Hospital, the only third-level children's hospital in El Salvador, 74% of the annual budget goes to pay salaries, leaving only 26% to pay for medications, electricity, and other resources [21]. According to Dr. Selva Sutter, Dean of the School of Public Health of a prestigious private university in San Salvador, “the government spends a lot in healthcare, but it doesn't do it efficiently” [21]. It is clear that the insufficient funding of public health care leads to exploitation of interns and residents in training, which puts patients at risk and jeopardizes the well-being of the trainees themselves. Medical educators and government agencies in charge of regulating the medical profession worldwide should develop programs that teach trainees and faculty to recognize the consequences of fatigue, sleep deprivation, and cognitive impairment, and implement strategies to counteract their deleterious effects. As Carolyn M. Clancy, Director of the United States Agency for Healthcare Research and Quality has stated, “hospitals [and medical schools] should take steps to ensure that interns and residents don't get fatigued to the point where they are providing unsafe care” [22].

I would suggest at least three specific steps that should be taken to reduce the work hours of physicians in training in El Salvador. First, the government should raise investment in health care or at least spend the available resources more efficiently, so that public hospitals could hire more personnel to handle the hospitals' work load. Hiring more personnel would allow a reduction of interns' work hours and an annual three week paid vacation for physicians in training, as mandated by the International Labour Organization [23]. More doctors and nurses means fewer shifts and fewer working hours, which could also mean fewer medical errors. Such errors are incredibly costly to hospitals—for example, medical errors cost the US US$17–US$29 billion each year [5].

Second, the job of every staff member should be redefined. For instance, interns should not be responsible for assisting nurses in taking a patient's blood pressure before the nurse administers furosemide, nor should interns be in charge of taking patients' vital signs or bringing urine samples to the lab.

Finally, if real change is to occur, the work hours of physicians in training in El Salvador and elsewhere must be regulated by law. For many years there has been talk in El Salvador of the need for reforms in the health care sector. Improving the working conditions of health care workers is a crucial aspect of these reforms [24].

## Supporting Information

Alternative Language Text S1 Spanish Translation of the Article by KRFT (56 KB DOC).
