# Supplementary material for: Excessive Work Hours of Physicians in Training in El Salvador: Putting Patients at Risk
Source: PLoS Med. 2007 Jul 17;4(7):e205. doi: 10.1371/journal.pmed.0040205 (PMC1914065; doi:10.1371/journal.pmed.0040205)
Supplement: Supplementary file 1 [file pmed.0040205.sd001.doc]

**Exceso de horas laborales de médicos en formación**

**en El Salvador: poniendo en riesgo a los pacientes**

**Kenneth R. Fernández Taylor**

Traducción del ingles por Mariana Taylor

Kenneth R. Fernández Taylor es un médico en formación de 8º año, Facultad de Ciencias de la Salud “Dr. Luis Edmundo Vásquez”, Universidad Dr. José Matías Delgado, La Libertad, El Salvador.

E-mail: [editor.signosvitales@gmail.com](mailto:editor.signosvitales@gmail.com)

*“Démosle emancipación al estudiante, démosle el tiempo y la oportunidad para el cultivo de su mente, para que en su preparación no sea un títere en manos de otros, sino un ser autosuficiente y reflexivo”.*

– Sir William Osler

Estudios recientes sobre las horas laborales excesivas de médicos en formación han mostrado que las jornadas laborales excesivas se relacionan con un creciente índice de errores médicos y eventos adversos. Estudios en residentes de cirugía han encontrado que el número de errores técnicos en el desempeño de habilidades quirúrgicas laparoscópicas simuladas se duplica luego de haber trabajado la noche previa, a diferencia de haber tenido una noche de sueño [1, 2]. Un estudio de internos trabajando en una unidad de cuidados intensivos encontró que se cometen más errores médicos graves cuando trabajan turnos frecuentes de 24 horas o más, que cuando trabajan turnos más cortos [3]. Un estudio nacional prospectivo de internos en los Estados Unidos encontró que los turnos de trabajo de larga duración (definidos como trabajo de 24 horas continuas como mínimo) se asocian con un alto riesgo de errores médicos graves, eventos adversos y fallas en la atención [4]. De acuerdo al Instituto de Medicina de los Estados Unidos [5], los errores médicos prevenibles son responsables de la muerte de hasta 98,000 personas en los Estados Unidos cada año, lo cual significa una tasa de mortalidad más alta que los accidentes de tránsito, el cáncer de mama, o el SIDA. A pesar de que no se sabe que cantidad de estos errores médicos fueron debido a la fatiga, el tema del exceso de horas laborales de médicos en formación se está convirtiendo en una prioridad de Salud Pública en muchos países alrededor del mundo.

En los Estados Unidos y Europa, la preocupación sobre los efectos que causan las horas excesivas de trabajo a la seguridad de los pacientes ha llevado a restricciones de las horas de trabajo bajo un marco legal [6-8], y más cambios están en camino [9]. Por ejemplo, la Directiva del Tiempo de Trabajo de Europa limita las horas de trabajo de médicos en formación a 58 horas por semana (48 para el 2009) [8]; no obstante, en muchos países, incluyendo El Salvador, no se han tomado medidas de ningún tipo para limitar las horas de trabajo de los médicos en formación. El actual máximo de 58 horas de trabajo por semana en Europa es menos de la mitad del tiempo que un médico en formación trabaja en El Salvador. De igual forma, muchos otros países Latinoamericanos también obligan a sus médicos en formación a trabajar horas excesivas. En el presente artículo, comparto mi experiencia personal de una típica semana de trabajo, discuto las consecuencias de las horas laborales excesivas y sugiero maneras de corregir el problema.

**Una semana de trabajo de 123 horas**

En El Salvador se llama internos a los médicos en formación que cursan el séptimo año de la carrera (de los ocho años de estudios de medicina). Un interno juega el doble rol de estudiante de medicina y empleado institucional, contratado por 365 días de trabajo obligatorio en un hospital nacional. Se comienza el 1ero. de enero y se finaliza el 31 de diciembre, trabajando siete días a la semana, con un mínimo de días programados para el descanso. Los internos ganan un salario mensual de $362 dólares americanos a cambio de más de 480 horas de trabajo. Por tanto, el salario por hora es de $0.75 centavos de dólar. Asimismo, muchos internos no reciben remuneración.

Si se es un interno, la hora de llegada al hospital es a las 5:00 a.m., y el trabajo concluye a las 5 o 6:00 p.m. los días de semana; los fines de semana se inicia a las 7:00 a.m., concluyendo a las 12:00 p.m. Se realiza un turno de 36 horas cada tercer día, lo cual significa que si se tiene turno un domingo, también se tendrá turno el miércoles y el sábado de esa misma semana, sin día libre alguno para recuperación en medio. Con suerte, probablemente se logre dormir una o dos horas durante un turno y tener entre 20 y 30 minutos para las comidas. Este régimen de trabajo llega a sumar 123 horas en una semana (una semana tiene 168 horas); únicamente de tres a cinco horas por semana son asignadas para actividades formativas. Además, la mayor parte del tiempo los médicos en formación (residentes e internos) trabajan sin ningún tipo de supervisión de parte de los médicos de staff. No hay duda alguna de porque las horas excesivas de trabajo de los médicos en formación han sido llamadas el tendón de Aquiles de la profesión médica [10].

**La carga de trabajo**

Las horas excesivas de trabajo de los médicos en formación en hospitales públicos se suman a una enorme carga laboral. Por ejemplo, el hospital nacional donde yo realicé mi internado es un hospital pequeño de segundo nivel con 222 camas, siendo este hospital el único en el departamento de La Libertad, cuya población es de más de 620,000 habitantes. En el 2006, 19,838 pacientes fueron hospitalizados; 44,671 emergencias y 4,367 partos fueron atendidos; y 5,542 cirugías fueron realizadas. Solamente hay 125 enfermeras/os, 70 médicos de staff, 40 residentes, y 44 internos para toda el área clínica del hospital, incluyendo las salas de operación y partos. Durante el turno nocturno de un residente de pediatría, por ejemplo, se encuentra solamente un residente, un interno y dos enfermeras asignadas para el área de hospitalización (alrededor de cincuenta niños). Durante la noche, puede haber más de veinte ingresos (admisiones), las cuales tendrán que ser cubiertas por el interno. El interno también debe tomar los signos vitales de cada paciente dos o tres veces como mínimo durante la noche, entre muchas otras responsabilidades.

Este enfoque inhumano hacia la formación de médicos ha sido presenciado por más de 75 estudiantes de medicina de la Federación Internacional de Asociaciones de Estudiantes de Medicina (IFMSA, por sus siglas en inglés) de los Estados Unidos, Europa y otros países. Dichos estudiantes han viajado a El Salvador como parte de un esquema de intercambio para estudiantes de medicina, y han criticado las condiciones perniciosas que sus colegas deben enfrentar en El Salvador.

**Consecuencias del exceso de horas laborales**

Existen muchas consecuencias lesivas vinculadas a las horas excesivas de trabajo de los médicos en formación, las cuales pueden ser divididas en consecuencias a corto y largo plazo. A corto plazo, dichas horas de trabajo conllevan a privación de sueño [11], también asociada con la fatiga, que atenta contra la seguridad de los pacientes [12]. La falta de sueño causa impedimentos neurocomportamentales como la disminución de la atención y la vigilancia, así como la inhabilidad de reconocer el grado propio de impedimento en el desempeño luego de un turno nocturno [13]. Dawson y Reid encontraron que los “niveles de fatiga relativamente moderados (luego de 17 horas de estado de vigilia) impiden el desempeño a un grado equivalente o mayor que el aceptado en los países occidentales industrializados para la intoxicación por alcohol” [14]. Las fallas en atención asociadas con la falta de sueño pueden ocurrir durante el trabajo directo con los pacientes [15]. Dichas fallas se relacionan con la inhibición de procesos cognitivos tales como la memoria y la intuición [16]. Los turnos de trabajo extensos realizados por los internos y residentes también se relacionan con un alto riesgo de accidentes automovilísticos [17].

A largo plazo, las horas excesivas de trabajo y la privación de sueño pueden llegar a incrementar el riesgo de burnout, ansiedad, depresión y suicidio [18]. El suicidio ha sido reportado como la segunda causa de muerte de los estudiantes de medicina [19]. En su estudio sobre cambios en el humor de internos a lo largo de su año de internado, Lisa Bellini y sus colegas encontraron que el entusiasmo al inicio del año pronto cedió ante la depresión, el enojo y la fatiga [20].

**Se necesita tomar acción**

El problema del exceso de horas laborales de los médicos en formación de El Salvador tiene raíces económicas y políticas que necesitan ser abordadas por políticos y especialistas de salud pública y políticas sanitarias. El Ministerio de Salud recibe recursos insuficientes de parte del gobierno para suplir las necesidades de la red pública de hospitales (donde consultan las personas de escasos recursos) negándole al sector salud la posición que merece en la agenda política nacional. Solamente el 1.62% del Producto Interno Bruto le es asignado al sector público de salud, que sirve al 85% de la población, mientras que al sector privado le es asignado el 6.38% [21]. Gran parte de los limitados recursos del sector público son invertidos en salarios. Por ejemplo, en el Hospital Nacional Benjamín Bloom, el único hospital de niños de tercer nivel en El Salvador, 74% del presupuesto anual es para pagar salarios, dejando solamente el 26% para cubrir costos de medicamentos, electricidad y otros recursos [21]. Según el Dr. Selva Sutter, decano de la Escuela de Salud Pública de una prestigiosa universidad privada en San Salvador, “el gobierno invierte mucho en salud, pero no lo hace eficientemente” [21].

Está claro que los fondos insuficientes asignados al sector público de salud conllevan a la explotación de los internos y residentes, lo cual pone en riesgo a los pacientes, y en juego el bienestar de los mismos médicos en formación. Las escuelas de medicina y agencias gubernamentales a cargo de regular la profesión médica a nivel mundial deberían desarrollar programas que eduquen a los médicos en formación y al personal de los hospitales a reconocer las consecuencias de la fatiga, privación de sueño e impedimento cognitivo. Asimismo, deben implementarse estrategias para revertir sus efectos nocivos. Además, como ha manifestado Carolyn M. Clancy, Directora de la Agencia Americana para la Investigación en Seguridad y Calidad Médica (AHRQ, por sus siglas en inglés) “los hospitales [y escuelas de medicina] deben tomar medidas para asegurar que los internos y residentes no experimenten un grado de fatiga al punto de otorgar cuidados inseguros” [22].

Por mi parte, sugeriría tres pasos específicos que deberían darse con el fin de reducir las horas laborales de los médicos en formación de El Salvador. Primero, el gobierno debe incrementar la inversión en el sector salud o al menos invertir los recursos disponibles de una manera eficiente, logrando que los hospitales públicos contraten más personal, mitigando la carga de trabajo. Contratar más personal permitiría una reducción de las horas laborales de los internos, además de una vacación anual pagada de tres semanas para los médicos en formación, como ha establecido la Organización Internacional del Trabajo [23]. Un mayor número de médicos y enfermeras significaría menos turnos y menos horas laborales, lo cual también puede significar menos errores médicos. Dichos errores son increíblemente costosos para los hospitales—por ejemplo, los errores médicos cuestan a los Estados Unidos entre $17 y $29 mil millones de dólares americanos cada año [5].

En segundo lugar, las responsabilidades de cada miembro del personal deben ser redefinidas. Por ejemplo, los internos no deberían estar a cargo de asistir a enfermeras/os para tomar la tensión arterial a los pacientes previo a la administración de furosemida por parte de ellas/os; del mismo modo, los internos no deberían cargar con la responsabilidad de tomar los signos vitales a los pacientes, ni de llevar muestras de orina al laboratorio.

Finalmente, para lograr que el cambio sea real, las horas laborales de los médicos en formación en El Salvador y otros países deben ser reguladas por la ley. En El Salvador se han llevado a cabo discusiones sobre la necesidad de reformas en el sector salud desde hace muchos años. La mejora de las condiciones laborales de los trabajadores del sector salud es un aspecto crucial de estas reformas [24].

**References**

1. Grantcharov TP, Bardram L, Funch-Jensen P, Rosenberg J (2001). Laparoscopic performance after one night on call in a surgical department: prospective study. BMJ;323:1222-1223.
2. Eastridge BJ, Hamilton EC, O'Keefe GE, Rege RV, Valentine RJ, (2003). Effect of sleep deprivation on the performance of simulated laparoscopic surgical skill. Am J Surg;186:169-174.
3. Landrigan CP, Rothschild JM, Cronin JW, Causal R, Burdick E et al. (2004). Effect of Reducing Interns' Work Hours on Serious Medical Errors in Intensive Care Units. New Engl J Med 351: 1838-1848.
4. Barger LK, Ayas NT, Cade BE, Cronin JW, Rosner B, et al. (2006) Impact of Extended-Duration Shifts on Medical Errors, Adverse Events, and Attentional Failures. PLoS Med 3(12): e487 [doi:10.1371/journal.pmed.0030487](http://dx.doi.org/10.1371/journal.pmed.0030487)
5. Kohn LT, Corrigan JM, Donaldson (eds). Comité on Quality of Health Care in America, Institute of Medicine. (1999). To Err is Human: Building a Safer Health System. Washington, DC: National Academy Press.
6. Report of the Council on Medical Education (2002) Resident physician working conditions (CME Report 9-1-02). Chicago: American Medical Association. Disponible: http://www.acgme.org/acWebsite/dutyHours/dh_wkgroupreport611.pdf
7. United Kingdom Department of Health (2003) Protecting staff; delivering services: Implementing the European Working Time Directive for doctors in training. Annex HSC2003/001. Disponible:: <http://www.dh.gov.uk/PublicationsAndStatistics/LettersAndCirculars/HealthServiceCirculars/HealthServiceCircularsArticle/fs/en?CONTENT_ID=4003588&chk=i2aOMz>.
8. Douglas, NJ. Sleep, performance and the European Working Time Directive. Clinical Medicine. 5(2):95-96, March/April 2005.
9. Dingel JD. US House of representatives Committee on Energy and Commerce, Washington, DC 20515 – 6115, One Hundred tenth Congress. March 29, 2007. Available: http://energycommerce.house.gov/Press_110/110-ltr.032907.HHS.Munier.pdf. Accessed 19 June 2007.
10. Leach, DC (2000). Residents’ Work Hours: The Achilles’Heel of the Profession? Academic Medicine. 75(12):1156-1157
11. Weinger, MB, Ancoli-Israel, S (2002). Sleep Deprivation and Clinical Performance. JAMA. 287(8):955-957.
12. Gaba, DM, Howard, SK (2002). Patient Safety: Fatigue among Clinicians and the Safety of Patients. New Engl J Med 347(16):1249-1255.
13. Arnedt JT, Owens J, Crouch M, Stahl J, Carskadon MA (2005). Neurobehavioral Performance of Residents After Heavy Night Call vs After Alcohol Ingestion. JAMA. 294(9):1025-1033.
14. Dawson D, Reid K. (1997). Fatigue, alcohol and performance impairment. Nature; 388:235-235.
15. Lockley SW, Cronin JW, Evans EE, Cade BE, Lee CJ et al (2004). Effect of Reducing Interns' Weekly Work Hours on Sleep and Attentional Failures. N Engl J Med. 351:1829 -1837.
16. Ellenbogen, Jeffrey M. (2005) Cognitive benefits of sleep and their loss due to sleep deprivation. Neurology. 64(7):E25-E27.
17. Barger LK, Cade BE, Ayas NT, Cronin JW, Rosner B, et al (2005). Extended Work Shifts and the Risk of Motor Vehicle Crashes among Interns. N Engl J Med 352:125-134.
18. Center C, Davis M, Detre T, Ford DE, Hansbrough W et al (2003). Confronting Depression and Suicide in Physicians: A Consensus Statement. JAMA. 289(23):3161-3166.
19. Roy A (1985). Suicide in doctors. Psychiatr Clin North Am. 8:377-387. Cited by: Hays, Lon R. MD et al (1996). Medical Student Suicide, 1989-1994. Am J Psychiatry. 153:553-555.
20. Bellini LM, Baime M, Shea JA (2002). Variation of Mood and Empathy During Internship. JAMA. 287(23):3143-3146.
21. Yamileth Caceres et al. Salarios por Medicinas. El Diario de Hoy. 17 de Agosto de 2006. Disponible: http://www.elsalvador.com/noticias/2006/08/17/nacional/nac10.asp
22. Clancy CM (2005). An Ongoing Discussion with the Readers of the Journal of Patient Safety. Journal of Patient Safety. 1(2):76-77.
23. International Labour Organization (1970) Holidays with pay convention (revised). Available: http://www.ilo.org/ilolex/cgi-lex/convde.pl?C132. Accessed 14 June 2007.
24. Organización Internacional del Trabajo (1999). Informe sobre las condiciones de empleo y de trabajo en el marco de las reformas del sector de la salud. Ginebra, 21-25 de septiembre de 1998. Disponible: <http://www.ilo.org/public/spanish/dialogue/sector/techmeet/jmhsr98/jmhsrr.htm>
